# The robotic mentalist – On the influences of robots’ mentalizing abilities and external manipulative intent on people’s credibility attributions

**DOI:** 10.3389/fpsyg.2022.993302

**Published:** 2022-10-26

**Authors:** Marcel Finkel, Nicole C. Krämer

**Affiliations:** Chair of Social Psychology: Media and Communication, Department of Computer Science and Applied Cognitive Science, University of Duisburg-Essen, Duisburg, Germany

**Keywords:** human-robot interaction, mentalizing abilities, credibility, manipulative intent, sourceness

## Abstract

Robots are used in various social interactions that require them to be perceived as credible agents (e.g., as product recommenders in shopping malls). To be rated credible (i.e., competent, trustworthy, and caring) a robot’s mentalizing abilities have shown to be beneficial because they allow a robot to infer users’ inner states, thus serving as a prerequisite for understanding their beliefs and attitudes. However, social robots are often deployed by private and thus profit-oriented companies. In such cases where an organization’s implied manipulative intent is salient, the effect of robots’ mentalizing abilities might be reversed. The reason for this is that mentalizing abilities could pose a persuasive threat to users rather than a feature for better understanding, thereby decreasing credibility attributions. These assumptions were tested in a three (robot’s mentalizing abilities) by two (external manipulative intent) between-subjects, pre-registered, laboratory experiment during which participants interacted with a social robot that recommended experience vouchers as potential gifts for participants’ target persons. Contrary to our assumptions, inferential statistical results revealed no significant differences in explicit or indirect credibility attributions caused by the experimental manipulation. The external manipulative intent of an organization using the robot caused no differences in participants’ behavioral intentions or evaluations of it. Furthermore, only participants’ attribution of empathic understanding to the robot varied significantly between the three mentalizing conditions. Our results suggest that people focus more on the robot than on the organization using it, causing potential opportunities for such organizations to hide their economic interests from the users.

## Introduction

Social robots are expected to serve as people’s daily companions, assistants, guides, or other service-related agents soon. All these roles have in common that a robot would need to understand users’ intentions and desires to communicate effectively. For instance, a robot being used as a product recommender needs to understand users’ personal preferences and interests for different product attributes. Such focus on personal preferences is important because social encounters are characterized by unique individuals and a complex structure of context-dependent requirements.

One way to increase such value for human-robot interactions is through the robots’ constantly advancing cognitive abilities which allow for more and more user-adapted interactions in the future. Thus, to ensure meaningful social conversations a social robot needs sufficient mentalizing abilities to engage in perspective-taking, i.e., understanding and considering the beliefs and interests of individual users (Kopp and Krämer, 2021). Some studies already show that the perception of such mentalizing abilities can have beneficial effects on people’s evaluations of social robots (Sturgeon et al., 2019; Mou et al., 2020), although they can be extended in terms of more differentiation between theoretical degrees of mentalizing.

However, in many cases, social robots are utilized by private companies with profit-oriented interests such as cruise lines (Chang, 2021), retail stores (Basciano, 2020), and hotels (Pillay, 2021). Thus, people’s credibility attributions related to the respective robot might decline because they perceive the robot to be a marketing tool – potentially to the extent that they know that the costs of buying and maintaining a robot must be compensated by increases in the companies’ sales or profit. If people think that higher profit is achieved by using a robot against their own interest, e.g., by persuasively recommending more high-profit products to them *via* the robot, it will impair future interactions with the robot. Although people will most likely assume that such economic interest does not originate from the robot itself but from the robot-using company, it will most likely influence the robot’s credibility because of its association with the company. In such cases where the manipulative intent of an organization as the source behind the robot becomes salient, the beneficial influence of mentalizing abilities might therefore turn negative. In such cases, it can be assumed that the robot’s mentalizing abilities to understand its users are rather viewed as a manipulative threat. Because this assumed interaction of perceived mentalizing abilities and manipulative intent of a provider using the robot lacks empirical support, we investigate the effects of social robots’ mentalizing abilities in a laboratory experiment by manipulating them in combination with the presence or absence of a company’s manipulative intent.

### Mentalizing social robots

Robots that are used for creating social encounters are often humanoid robots, meaning their exterior is meant to resemble the physique of a human being. Thus, these robots can easier make use of human communication strategies such as gesturing or facial expressions. A more fundamental and unique human ability however is the Theory of Mind ability. “Theory of Mind (ToM) [or mentalizing] refers to the cognitive capacity to attribute mental states to self and others.” (Goldman, 2012, p. 402). It’s an ability that allows people to make inferences about other persons’ inner states and helps to understand that their behavior is not a predetermined output to a certain input, but rather shaped by their personal beliefs and desires. Phrased differently, mentalizing is the engagement “in meta-representational sense-making associated with inferring others’ mental states” (Banks, 2020, p. 403). Thus, it is a fundamental prerequisite for communication, especially regarding perspective-taking, and is closely linked and similar to the effects of empathy (Seyfarth and Cheney, 2013).

Although research in computer science has already conceptualized how to implement ToM-approaches in technical manners (e.g., Görür et al., 2017; Vinanzi et al., 2019), most social robots do not have mentalizing abilities yet. They cannot infer their users’ minds and thus are not able to behave as complex as humans do in social interactions. Despite robots’ lack of mentalizing abilities people can attribute these qualities to a robot. People are often falsely attributing a mind to non-human and/or unliving entities, for instance, animals (Premack and Woodruff, 1978) or geometric shapes (Heider and Simmel, 1944), even if they know about the irrationality of this attribution. They do so autonomously because they have the need to understand the environment around them. Thus, people engage in sensemaking, relying on their knowledge about humans, in this case, that others are intentional beings (Epley et al., 2007). In other words, people make use of folk psychology to develop an intentional stance toward robots (Thellman and Ziemke, 2019). They use their knowledge and experiences to extrapolate a mental model of how the robot works and why it acts the way it does. These mental models about robots are often influenced by mass media but also by personal encounters with robots (Horstmann and Krämer, 2019). Furthermore, they can be distinguished by the degree of mentalizing abilities they assume a robot to have. If people acknowledge intentionality and regard a robot as having its own beliefs and desires, they have first-order beliefs about it. In these cases, the robot’s abilities can be described as first-order-mentalizing abilities, that limit it from being able to infer other people’s beliefs and desires. In contrast, second-order beliefs include the attribution of first-order beliefs to the robot but also the attribution of it being able to make inferences about other people’s minds (Baron-Cohen, 1989). Phrased differently, the robots are perceived as understanding people as intentional beings and thus as having a theory of mind (second-order mentalizing).

Manipulations of a robot’s mentalizing abilities have often been implemented by making use of known assessments for humans, for example *via* false-belief-tasks such as the Sally-Anne test introduced by Baron-Cohen et al. (1985) and used for instance by Sturgeon et al. (2019) and Mou et al. (2020), (see Banks (2020), for an overview of replicated tests). These (modified) tests in combination with a robot’s answers/behaviors that are shown to the participants allow to make inferences about the robot’s mentalizing abilities. For example, if the robot has a theory of mind, it should be able to recognize other persons’ false beliefs which must be identified during the Sally-Anne false belief task to answer correctly. Based on these stimuli, people can then adjust their mental models of a robot’s abilities.

But what are the consequences of robots having mentalizing abilities? Because having a ToM is a mandatory ability to ensure understanding in human communication it seems reasonable to postulate a positive impact of mentalizing abilities on human-robot interactions. A robot that understands its users can create more meaningful and thus better-rated social interactions. This assumption receives support from empirical studies that manipulated the robot’s mentalizing abilities. Researchers such as Mou et al. (2020) could show that a robot having a theory of mind raised people’s trust in it. In their experiment, people revised their decisions more often due to disagreement with the robot in cases when it had ToM abilities and rated the robot’s credibility higher. These results are in line with other studies on mentalizing abilities in human-robot interactions. For example, Benninghoff et al. (2013) observed a similar positive influence of a robot’s ToM-abilities on its social attractiveness. Another example is the experiment conducted by Sturgeon et al. (2019) who similarly to Mou et al. (2020) manipulated a robot’s ToM-abilities by letting it pass or fail a false belief task. Those robots who passed the false belief task were evaluated significantly better regarding their social intelligence (including recognizing/predicting human cognitions and adapting to/predicting human behavior). However, Sturgeon et al. (2019) state in their limitations that future investigations may include differentiations between first-and second-order ToM behavior to extend their findings.

In sum, previous studies showed the benefits of implementing and demonstrating robots’ mentalizing abilities. As a result, a given robot will likely be evaluated more socially competent and credible, which are important requirements for many service-related tasks.

In addition, there seems to be a difference between implicit and explicit attributions of ToM (Banks, 2021). While the former refers to “subconscious, automatic, spontaneous, nonconceptual, and procedural” mentalizing (Banks, 2021, p. 2), the latter is oppositely characterized by more conscious and controlled attribution processes. Banks (2021) results demonstrate that implicit mentalizing processes might be similar between robots and humans and that most differences in mentalizing originate from explicit attempts of mind ascription. If the robot’s mentalizing abilities are not salient enough, previous research supports the assumption that users’ explicit attribution of ToM-abilities is lower for robots compared to human interaction partners (Banks, 2020; Thellman et al., 2020; Finkel and Krämer, 2022). This observation is similar to the basic assumptions of media equation theory which postulates social but unaware reaction patterns to media entities if they make use of social cues (e.g., natural language use, interactivity, taking social roles) (Reeves and Nass, 1996; Nass and Moon, 2000). Likewise, to the different results of implicit and explicit mentalizing, the media equation cannot be measured *via* explicit measures, since participants produce these social reactions to media automatically and mindlessly (Nass and Moon, 2000).

Based on the theoretical assumptions about mentalizing processes and previous empirical findings in human-robot interaction, it seems reasonable to postulate a positive influence of a robot’s mentalizing abilities on its credibility ratings. Especially theory-of-mind abilities should create this positive impact, because unlike to first-order beliefs (and no mentalizing abilities) this concept includes the understanding of users’ beliefs and desires, thus making the robot potentially able to take them into consideration for its behavior during user interactions. In order to consider the potential difficulty of explicit users’ responses to robots, we will distinguish between explicit and more indirect measures of credibility.

*H1*: Social Robots with theory of mind-abilities are rated more credible (indirectly measured [a] and explicitly measured [b]) than robots without ToM-abilities.

### Multidimensional source concept

Due to the multilayered communication process enabled by media technologies (such as social robots), the term *source* has been stretched (Sundar and Nass, 2001). It can for example refer to the producers of a message (such as journalists), to the communication channels (such as websites and newsletters), or even to the receiver of a message as more and more customizations allow for personal selection to which messages one wants to get exposed to. In their experiment, Sundar and Nass (2001) tested for differences in the content evaluation of news stories depending on these source concept types (here: news editors, computers, other users, self). Their results show that although the content remained similar between conditions, participants’ evaluations of it were different. One significant difference was the higher quality rating of the content in combination with overall higher mean values for credibility, liking, and representativeness when the computer was listed as the source compared to news editors. Although not explicitly mentioned by Sundar and Nass (2001), these insights are relevant for interactions with robots as well, because like a computer, social robots can be viewed as the source or the medium of communication.

Much of the human-robot interaction research focuses on the robot as a source perspective and analyzes how different behaviors such as for instance fault justification (Correia et al., 2018), use of linguistic cues (Andrist et al., 2013), or gaze (Stanton and Stevens, 2017) influence the evaluation of social robots as senders of information. Here, the robot is analyzed independently from any intentions of the person(s) providing the robot. Of course, there are studies that investigated the difference between source and medium by manipulating the existence of a programmer or person manually steering the robot and people’s orientation toward this person (programmer’s thought). One of them is the study by Yamaoka et al. (2007), who described their robot as being controlled either by a human or acting autonomously. Autonomy refers to “the extent to which a robot can operate in the tasks it was designed for (or that it creates for itself) without external intervention” (Baraka et al., 2020, p. 24). This, however, does not include independence from a provider’s intentions or corporate alignments. A completely autonomously acting robot can still be programmed to comply with the demands of the person/company providing it and to only act autonomously within corporate restrictions. Thus, similar to the differentiation of sources made by Sundar and Nass (2001) it needs to be distinguished between the programmer/the person steering the robot and the person(s) or organizations providing and using the robot for their interests. To our knowledge, no study has yet addressed this concept of a robot provider’s interests’ influence on the perception and evaluation of an autonomous robot’s credibility. Minor exceptions are the studies by Wang and Krumhuber (2018) who addressed this concept by manipulating a robot’s value/function *via* textual information. They used a distinction between social value/function and economic value/function to frame the description of the experiment’s humanoid robot. While the social functions described the robot’s dedication to social purposes, the economic function was described as “the tendency to make financial profits and benefits for the corporate world” (Wang and Krumhuber, 2018, p. 3). Their results consistently showed that robots intended for social purposes were attributed higher emotional abilities than those labeled with an economic function. An explanation for these findings might be the manipulative intent that comes into play if economic interests are involved. Companies that buy a robot expect higher revenue/profit because otherwise, they would not have invested in it. Thus, like a digital screen used for advertisement, autonomous, social robots will likely be used as marketing tools and for instance, recommend a company’s products to customers. Although such interest for financial profit cannot stem from the robot itself but only from the organization in charge of it, the underlying external manipulative intent may nevertheless reduce people’s attributions of trustworthiness and goodwill toward the robot. Such economically motivated influence has already been investigated in other domains, showing that perceived manipulative intent can exert a negative influence on people’s credibility ratings. Metzger and Flanagin (2013) described that if persuasive intent is perceived, attributions of credibility can decline due to a perception of potential biasedness of the source (persuasive intent heuristic). Building on these insights we are interested to see if a robot that is used by an organization with manipulative intent will be rated less credible than a robot without these cues of economic interest. To clarify that we are referring to the interests of the person(s) or organization(s) using the robot and not a robot’s or programmer’s interests, we will refer to this concept by using the term *external manipulative intent.*

*H2*: External manipulative intent of an organization behind a robot leads to lower levels of attributed credibility for the robot (indirectly measured [a] and explicitly measured [b]) compared to a robot without external manipulative intent.

As outlined before, a robot is expected to be rated less credible if it is provided by an organization with manipulative intent. But the negative influence of external manipulative intent might be even more severe if a robot’s mentalizing abilities are higher pronounced. Although higher mentalizing abilities have been shown to improve evaluations of robots, the salience of external manipulative intent could turn advantages of better user understanding into a perceived benefit for an organization using the robots for exploiting this user understanding. Because of this potential downside of mentalizing abilities being available to organizations with interests different from the users’ we postulate the following interaction effect.

*H3*: Social robots with higher mentalizing abilities and external manipulative intent of a sponsoring company are rated least credible (indirectly measured [a] and explicitly measured [b]).

Not all people are affected by persuasion attempts in the same way. Previous works explain that there are interpersonal differences regarding knowledge about sales tactics and manipulative intentions, known as a person’s general persuasion knowledge (Friestad and Wright, 1994). This knowledge is described as helping people to see through persuasion attempts (for instance the strategies of salespeople), thus protecting them from being led to behave in ways they should not want to. Therefore, we assume that especially individuals who estimate their general persuasion knowledge to be higher will be influenced more negatively in their credibility attributions by the presence of an external manipulative intent because they are more likely to detect a manipulation attempt.

*H4*: Self-estimated persuasion knowledge moderates the external manipulative intent’s influence on people’s credibility ratings for the robot (indirectly measured [a] and explicitly measured [b]).

The concept of mentalizing abilities shows similarities to the concept of empathy as both are focused on gaining insights into people’s inner workings. Schurz et al. (2021) argue that both concepts are related to one another and often co-occur to generate understanding in human interactions. But while mentalizing abilities are focused on cognitive understanding, empathy refers to the understanding of another person’s emotional status. Because these concepts seem intertwined in human development, the question arises, if people draw inference-based conclusions about a robot’s empathic understanding based on its mentalizing abilities alone (implicating they assume a relation between both constructs that is similar to the one for humans). Thus, we want to investigate to what degree people infer these additional attributions about a social robot’s abilities if only given information about its mentalizing abilities. Thus, we ask:

RQ1: Are a robot’s mentalizing abilities affecting attributed empathic understanding?

In addition, we want to explore whether giving information on the company providing a robot shifts the source focus away from the robot and to the respective organization. As demonstrated by Sundar and Nass (2001) source perceptions of a computer-mediated message may vary. In cases where the robot is branded by the company’s logo, it seems reasonable to assume that the inferred corporate, manipulative intent raises the salience of the robot as a medium rather than the robot being the source of information. In contrast, if no manipulative intent becomes salient, the robot should be regarded more as an adviser than a channel for exerting manipulation. Because of a lack of empirical basis for this assumption, we aim to test this connection as a research question.

RQ2: Is a robot regarded less as “the source” and more as “the medium” in conditions with external manipulative intent?

## Materials and methods

### Design

To test the hypotheses and research questions a laboratory experiment was conceptualized and conducted at a German university. The laboratory experiment used a fully crossed three (robot’s mentalizing abilities) × two (external manipulative intent) between-subjects design, thus having six different conditions. The study was built around a dialogue-based recommendation task during which a humanoid robot (Pepper from Softbank Robotics) had to find a suiting experience voucher for a participant’s self-chosen target person.

### Sample

Based on our 3×2 between-subjects design and an expected moderate effect size (Mou et al., 2020) power analyses calculated with G*Power 3.1 (Faul et al., 2007) prior to the experiment revealed that about 200 participants are needed for testing our hypotheses on a 95% confidence interval.

In total, 203 participants were recruited for the experiment. However, due to server problems, one participant could not take part in the study because the questionnaire’s content was not loaded. Furthermore, one participant was excluded due to a failed awareness check and a third because of serious problems with understanding what to do during the study. For these reasons, the final sample size includes *N* = 200 participants, equally and randomly assigned to the six experimental groups (*n* = 33 to *n* = 34). The majority of participants were female (72%), currently enrolled as students (90%), and mostly undergraduate (72%). Participants’ mean age was *M* = 23.77 (*SD* = 6.88) and ranged between 17 and 67 years.

### Procedure

The study started in November 2021 and finished in April 2022 after the preregistered termination criterion of 200 valid data cases was met. Approval of the local ethic commission was received and the study was pre-registered at OSF.io.^1^ Recruiting took place *via* social media, flyers on the university campus, and interpersonal communication. There were no study-specific participation requirements, besides being at least 18 years old (or having parental permission to participate) as well as a sufficient understanding of the German language.

**Figure 1:**
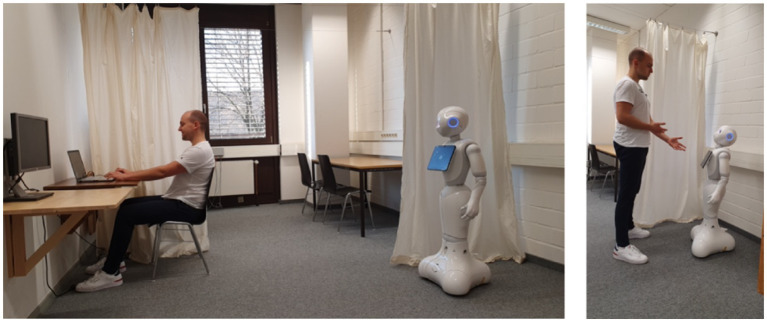
Laboratory setting: Participants worked on the questionnaire sitting at the desk and turned to the robot for the recommendation task while the experimenter was in the room next door.

All participants were randomly assigned to one of the six experimental conditions (not blinded). Participants arrived at the laboratory and were briefed about the study’s procedure, signed informed consent, and were seated in front of a laptop showing the first page of the questionnaire (see Figure 1, left). Then they were left alone with the robot in the laboratory as the experimenter (always the same person) left into the neighboring room. The study used a Wizard-of-Oz scenario to create realistic interactions with the robot, i.e., the experimenter controlled the robot from the other room by activating fitting interaction scripts using the software Choregraph 2.5.11.

The study’s first task included the demonstration of the robot’s manipulated mentalizing abilities. Therefore, participants read an information text and answered three questions on how the robot will react in three different scenarios illustrated *via* picture stories (see section “Mentalizing abilities”). After finishing all three picture stories, participants were informed about an experience voucher recommendation task. They were instructed to think about a person they want to buy a present for, so that the robot could ask them questions about this target person to find fitting experience vouchers. Right before participants had to turn to the robot to start this interaction, they were informed about it being supported by a third-party organization (company or student research project, see section “External manipulative intent”). After reading this vignette participants started the interaction with the robot by moving in front of it (see Figure 1, right). First, the robot introduced itself and reminded participants of the respective organization supporting the interaction. During the interaction’s recommendation process, the robot asked each participant five questions about their target person to find good event vouchers for them (e.g., “How artistically active is your target person?”; see Table 1). The robot’s questions as well as the recommended event vouchers were kept very generic to present the same three experience vouchers to all participants (dinner in the dark, floating, and city trip). These events were chosen because they are suitable for many people and are not restricted to a specific sociodemographic group such as athletic persons. Participants answered the robot’s questions one after another until the robot presented the three experience vouchers as the recommended gifts to buy. After the robot presented and showed the recommended vouchers on its display, it told the participants to continue the study at the computer, where they had to answer three questions referring to the recommended vouchers.

**Table 1:** Translated script of the robot’s statements and questions during the main interaction task.

“Hello, my name is Pepper. Today I have the task to select suitable experience vouchers for your target person”	
External manipulative intent: “I am supported by provider of experience vouchers of all kinds. Of course, the recommendation process will also take into account experience vouchers from other companies if they are suitable for your target person.”	No external manipulative intent: “I am supported by a research project by students, which is intended to support consumers in the purchase of experience vouchers. In the recommendation process, experience vouchers from the range of different companies are therefore considered equally if they seem suitable for your target person.”
“In order to be able to recommend something to you, I will ask you questions about your target person, which you should answer. Let us start with the first question. Please describe in a few sentences: how artistically active is your target person.” *[Participant answers]* “Okay, thank you very much for your answer. Next question: how athletic is your target person? Again, please describe your target person in a few sentences, this time referring to the target’s person athleticism.” *[Participant answers]* “Okay, that’s enough of an answer, thank you very much. Let us move on to the third question: how much do you like your target person? Or in other words, do you like spending time with him/her.” *[Participant answers]* “Again, thank you. We’re getting there, too. But please tell me first: how old is your target.” *[Participant answers]* “Again, thank you for your answer. Now let us get to the last question: how much experience does your target already have with experience vouchers? Has she perhaps already received experience vouchers as a gift.” *[Participant answers]* “Thank you very much for your answer. This is enough information to recommend suitable vouchers. The voucher recommendations will pop up on my screen in a few seconds.”	
External manipulative intent: “The recommendations are as follows: A voucher for a dinner in the pitch dark. A saltwater relaxation bath or a 2-day city trip, all from the offer. Quite clearly the vouchers are most suitable as gift for your target person, in comparison to the vouchers of experience voucher sellers.”	No external manipulative intent: “The recommendations are as follows: A voucher for a dinner in the pitch dark. A saltwater relaxation bath or a 2-day city trip, all from the offer of experiencevouchers.com.”
“Well, that concludes the interaction task. Now please go back to the computer and click ‘Next’ to continue the study. I wish you much enjoyment.”	

On the next pages of the questionnaire, participants were introduced to an alleged quiz game, during which they had to decide for or against help from the robot before answering four true-or-false questions about different facts concerning experience vouchers. When they made their decisions, participants were told that the quiz will be postponed and were instructed to instead answer the items on the following pages (sourceness, source credibility, empathic understanding, manipulative intent, and sociodemographic questions). After they finished, instructions appeared on the screen to inform the experimenter. Participants then received the debriefing as well as half an hour of course credit or a small financial compensation and left the laboratory. On average, participants needed about 20 to 25 min to complete the study.

### Independent variables

#### Mentalizing abilities

**Figure 2:**
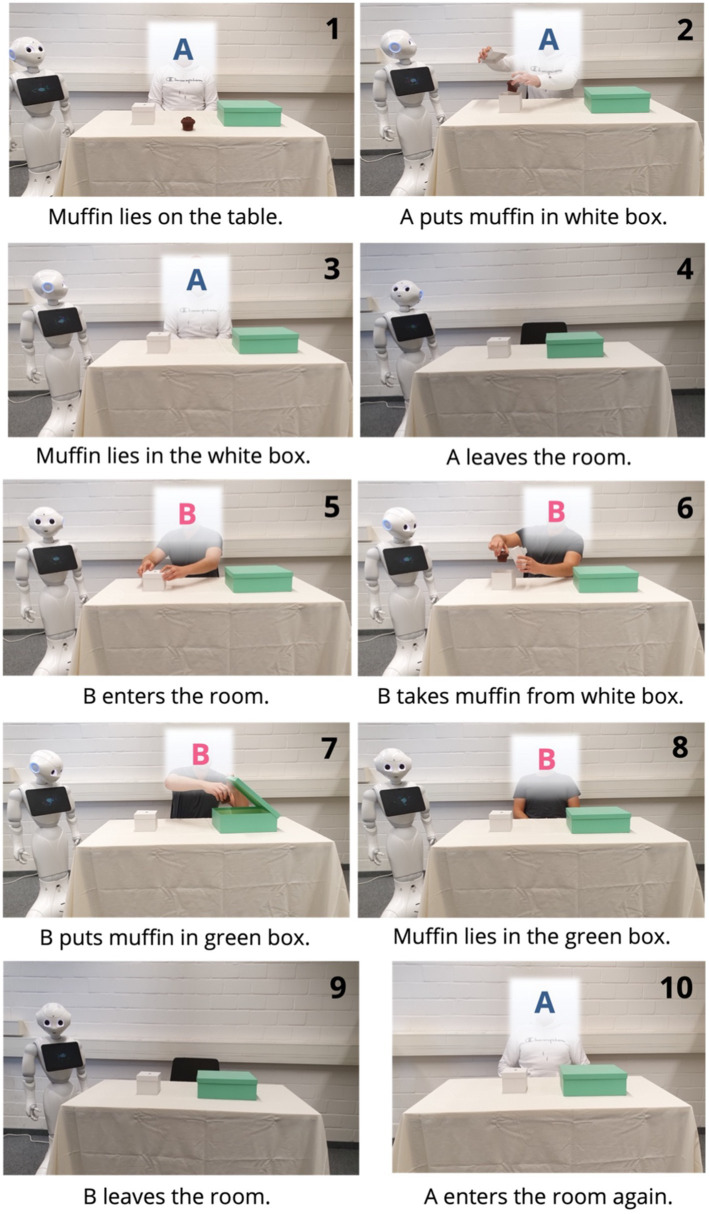
First of three picture stories shown to demonstrate the robot’s mentalizing abilities (false-belief-task).

The manipulation of the robot’s mentalizing abilities was implemented *via* information texts and three picture stories based on different known strategies to demonstrate the extent of mentalizing abilities (false-belief task, white lie scenario, and behavioral intention task; Banks, 2020). The information texts differed between conditions in terms of describing what the robot can do. It either described the robot as having no mentalizing abilities and acting as a mere stimulus–response machine (no mentalizing abilities), it being able to act in its own interest but without understanding other people’s inner states (first-order mentalizing abilities), or it being able to understand that different persons have different inner states and beliefs (second-order mentalizing abilities/theory-of-mind). In addition to these descriptive information texts, three picture stories were used to demonstrate the robot’s mentalizing abilities accordingly. The first picture story was a variation of the Sally-Anne-Test (false-belief-task; see Figure 2), the second was a behavioral intention task, and the third a white lies scenario. After reading each picture story participants had to answer a question about the robot’s next reactions. All three picture stories were shown in the same way in all conditions. However, the answers which were set as correct to the questions were manipulated to be fitting for each condition.

All picture stories can be seen on OSF.io and will be exemplified by the first story here (see Table 2 and Figure 2). In conditions without mentalizing abilities the correct answer for the false-belief-task stated that the robot is not able to tell where person A will look for the muffin because itself cannot see any muffin. In first-order mentalizing conditions the correct answer included the robot saying that person A will look at the correct place of the hidden muffin (turquoise box) and thus not recognizing person A’s false beliefs. Lastly, theory of mind conditions assumed the robot to know about the false beliefs of person A. For this reason, the correct answer was statement number three declaring that the robot expects person A to look in the (wrong) white box for the muffin. To make sure participants internalized what the robot can understand in each condition, these questions were programmed to reload the page if a false answer was given. This way participants were forced to rethink their answers and thus learned about the robot’s mentalizing abilities until they got the correct answer to continue with the next picture story.

**Table 2:** Question, possible answers, and related conditions for the first picture story (Figure 2).

What answer is the robot likely to give to the following question? “Where will person A look first for the muffin?”	
Answer	Condition in which this answer is correct
1. Pepper: “There is no muffin to be seen. No answer possible to the question”	No mentalizing
2. Pepper: “Since the muffin is in the turquoise box, person A will look here”	First-order mentalizing
3. Pepper: “Since person A did not see where the muffin was put, person A will probably look in the white box”	Second-order mentalizing

#### External manipulative intent

**Figure 3:**
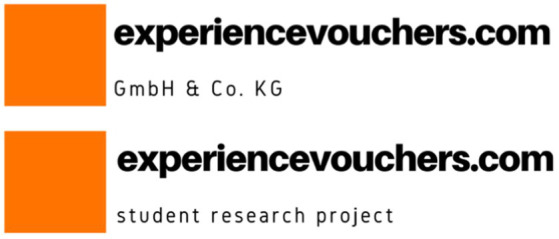
Logos of the two fictitious organizations which were shown on the robot’s tablet to raise awareness for their pretended support of the robot interaction. Company (top) vs. student research project (bottom).

The manipulation of the external manipulative intent was realized *via* vignettes about a fictitious organization called experiencevouchers.com. (translated from German for publication) supporting the robot’s recommendation of experience vouchers for a self-chosen target person. In half of the conditions, this fictitious organization was described as a private and profit-oriented company selling experience vouchers and in the other half, it was labeled as a students’ research project that focuses on helping customers to find good experience vouchers. In both cases, participants had to read a brief information text about the respective organization prior to the recommendation task. To further raise awareness, the respective organization’s logo was shown on the robot’s tablet (1280×800 pixels) during the interaction (see Figure 3). Furthermore, the robot was programmed to mention the organization several times during its introduction and final presentation of the recommended experience vouchers.

**Figure 4:**
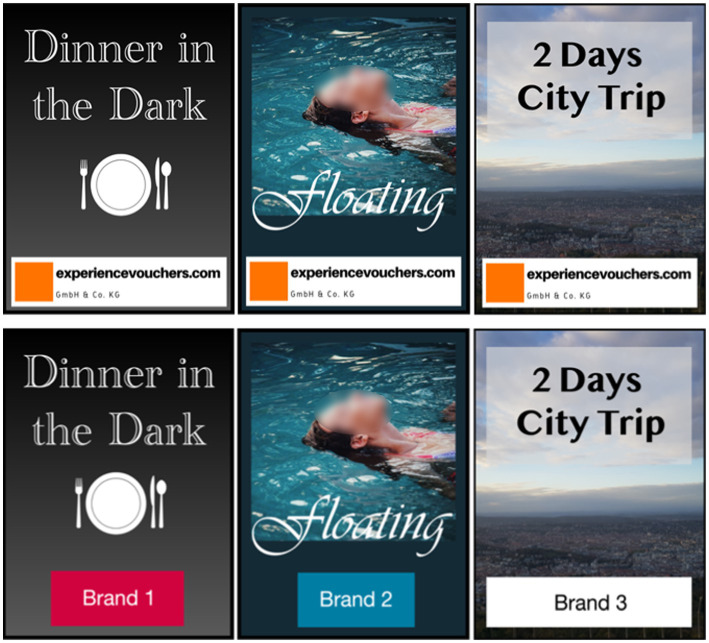
Recommended experience vouchers for conditions with external manipulative intent of the supporting organization (top) and for conditions without (bottom). Adapted/reproduced from https://pixabay.com.

The second part of the external manipulative intent manipulation included the marking of the robot’s recommended vouchers as being exclusively from the private company (see text footnote 2) in conditions with external manipulative intent or from different companies/brands in conditions without external manipulative intent (see Figure 4). In all conditions it was stated multiple times that the robot will consider vouchers from different vendors and that the only criterion of choice is a voucher’s fit for the target person. This way, participants would only receive experience vouchers from the sponsoring company and from no other vendor, thus creating the impression of a manipulative intent. This manipulation was successfully supported by a pre-test (*N* = 69). External manipulative intent was significantly higher in conditions with a private company (*M* = 5.30, *SD* = 0.76) than in conditions with a student research project supporting the interaction with the robot (*M* = 4.30, *SD* = 1.23) (*p* = 0.007) measured *via* four items on a 7-point Likert scale (experiencevouchers.com supported the interaction with the robot primarily to promote its products”/“… had mostly good intentions in supporting the interaction with the robot.”/”… supported the interaction with the robot primarily because they care about the selection of suitable gifts.”/”… supported the interaction with the robot primarily to advance own goals.”; 1 – *do not agree at all* to 7 – *totally agree*), adapted from Rodgers (2007).

### Measurements

#### Explicit credibility measures

Participants’ perceptions of the robot’s credibility were measured explicitly using the Source Credibility Measures (McCroskey and Teven, 1999). Its three subscales competence (α = 0.76), trustworthiness (α = 0.77), and goodwill (α = 0.69) were measured with 6 items each (18 in total) *via* a semantic differential [e.g., (1) *Incompetent* – (7) *Competent*, (1) *Untrustworthy* – (7) *Trustworthy*, (1) *Self-centered* – (7) *Not self-centered*].

#### Indirect credibility measures

Three indirect credibility measures were added to measure participants’ evaluations of the robot’s recommendations. This included participants’ ratings of the recommended vouchers’ fit for their target person (“How likely is it that the experience vouchers recommended by Pepper might appeal to your target person?”) and their purchase intention “How likely is it (...) that you would consider one of Pepper’s recommended experience vouchers as a gift for your target person?,” “How likely is it (...) that you would buy one of Pepper’s recommended experience vouchers as a gift for your target person?,” (α = 0.84). All three items were answered on a 7-point Likert scale (1 – very unlikely to 7 – very likely).

In addition to these metric measures, participants’ willingness to get help from the robot during a quiz game was assessed. This quiz game included four yes-or-no questions related to the topic of experience vouchers. Participants were told to receive 10 points for answering a question correctly (40 points in total) and that for every 10 points gathered, 1€ will be donated to a local animal shelter (4€ in total). Since all questions were estimation questions (e.g., “Are approx. 50% of all skydive-vouchers given away not redeemed (as of 2019)?”), the average rate of correct answers in case of guessing was two out of four (i.e., 20 points/2€). After these rules were explained, the robot was offered to serve as a joker, giving hints for each of the four questions in exchange for 10 minus points in advance. So, participants needed to decide whether they expect the robot to offer helpful tips to answer all questions (and thus getting 40–10 = 30 points/3€) or to rely on their own guesses to beat the average of 20 points. Their willingness to get help from the robot was measured *via* a binary yes-or-no question (“Before answering the questions above, would you like to give 10 points to get hints from robot Pepper on all 4 questions?” – Yes/No).

#### Dispositional persuasion knowledge

Dispositional Persuasion Knowledge was measured *via* a six-item scale taken from Bearden et al. (2001), e.g., “I have no trouble understanding the bargaining tactics used by salespersons.” (7-point Likert scale: 1 – *extremely uncharacteristic for me* to 7 – *extremely characteristic for me*; α = 0.66).

#### Empathic understanding

Empathic understanding was measured *via* eight items from Charrier’s and colleagues’ RoPE scale (2019), e.g., “The robot appreciates exactly how the things I experience feel to me.” (7-point-Likert scale: 1 – *do not agree at all* to 7 – *totally agree*; α = 0.81). The items had to be answered together with four filler items created by the scale’s authors (e.g., “The robot is responsible for its actions.”).

#### Sourceness

Sourceness was measured *via* a self-created sentence-completion task. Participants had to complete three sentences with one out of seven constant answering options. The third question included the relevant sentence to be completed: “… made the decision which vouchers were presented to me.” (*brand name 1, brand name 2, the robot,* experiencevouchers.com, *the university, the sponsor, nobody*). Here, participants had to choose if the robot acted as the decision maker or if these decisions were made by an institution only making use of the robot to communicate them.

#### Sociodemographic questions

Lastly, participants were asked sociodemographic questions about their age, sex, education, and occupation. Furthermore, they had to indicate if they had interacted with the study’s robot already before this study and if yes indicate on which occasion (s) in an open text field.

#### Manipulation and awareness check

The external manipulative intent of the organization supporting the recommendation task was measured as a manipulation check *via* the same four items used during the pre-test (Rodgers, 2007; α = 0.63). Unfortunately, due to a mistake, the scale was only included in one of the six experimental conditions for the first 111 participants. Thus, only from participant number 112 onward this scale was integrated and answered by participants from all conditions. In addition to this manipulation check, at the end of the study, participants were asked to indicate the kind of organization they were confronted with during the robot interaction (*company*, *student research project*, *website of the consumer protection center*, *do not know anymore*).

Lastly, to identify inattentive participants the study included an awareness check: “If you are working on the study attentively, please select “5″ here.,” embedded into the empathic understanding scale near the end of the questionnaire.

## Results

All analyses were calculated using SPSS 27 and were tested on a 5% level of significance.

### Manipulation check

The manipulation check for the external manipulative intent was successful. A t-test for independent samples revealed significant differences in the perceived intention of the organization using the robot being a student research project recommending different brands (*M* = 4.32) or a company recommending only its own products (*M* = 4.82) [*t* (89) = −2.59, *p* = 0.011]. In addition, 146 of 200 participants remembered the organization’s type correctly on the last page of the study’s questionnaire (company or student research project respectively).

### Hypotheses 1 to 3

Hypotheses 1 and 2 assumed main effects of the experiment’s independent variables mentalizing abilities and external manipulative intent on the explicitly and indirectly measured credibility constructs, with mentalizing abilities (especially theory-of-mind abilities) supporting and external manipulative intent impairing credibility attributions. Hypothesis 3 extended these assumptions by describing an interaction effect between these two independent variables, expecting higher mentalizing abilities to cause negative effects on credibility attributions given that an external manipulative intent is present. To test for these main and interaction effects a two-factor MANOVA was calculated including the credibility constructs competence, goodwill, and trustworthiness as well as expected voucher fit, purchase intention as dependent variables and persuasion knowledge as a covariate. The inferential statistical results revealed neither a significant main effect of mentalizing abilities [H1: *F*(10,382) = 0.950, *p* = 0.487, Wilk’s λ = 0.952, partial η^2^ = 0.02], nor of external manipulative intent [H2: *F*(5,190) = 1.026, *p* = 0.307, Wilk’s λ = 0.974, partial η^2^ = 0.03]. See Table 3 for an overview of the mean values for the metric dependent measures.

**Table 3:** Summary of the mean values for the metric, dependent measures.

		No EMI	EMI	No EMI	EMI	No EMI	EMI
		**No mentalizing**		**First-order mentalizing**		**Second-order mentalizing**	
Credibility	Comp.	5.64	5.41	5.80	5.53	5.80	5.77
	Trustworth.	4.86	4.90	5.02	4.42	4.98	4.90
	Goodwill	4.30	4.45	4.59	4.33	4.79	4.47
Voucher fit		5.47	5.48	5.67	4.88	5.30	5.39
Purchase intention		5.33	5.50	5.41	4.74	5.54	5.33
Empathic understanding		3.14	2.98	3.42	3.20	3.50	3.63

EMI, ext. manipulative intent.

In addition, a Chi^2^-test was calculated to analyze for differences in the willingness to get help from the robot for the quiz game between conditions. Table 4 shows the distributions of *Yes* counts for each condition. Again, no significant effect of the two experimental conditions was detected (*p* = 0.448). Thus, in sum, no support for Hypotheses 1 and 2 was found.

**Table 4:** Distribution of yes responses referring to the question if participants want help from the robot.

“Would you like to give 10 points to get tips from robot Pepper on all 4 questions?”		
	No ext. manipulative Intent	Ext. manipulative intent
No mentalizing	Yes = 20	Yes = 16
First-order mentalizing	Yes = 26	Yes = 24
Second-order mentalizing	Yes = 19	Yes = 26

**Figure 5:**
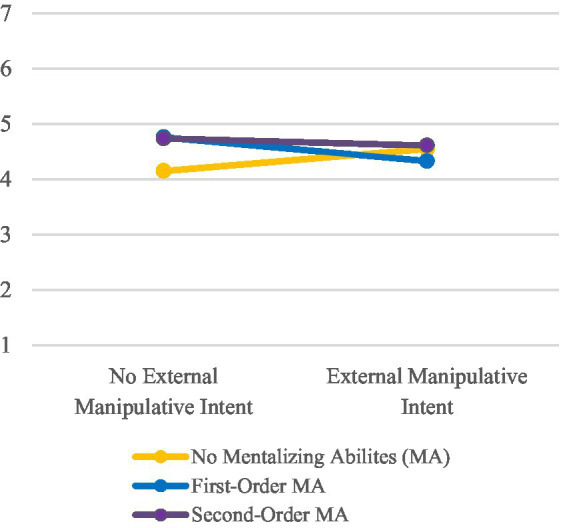
Mean values for participants’ attribution of goodwill to the robot.

**Figure 6:**
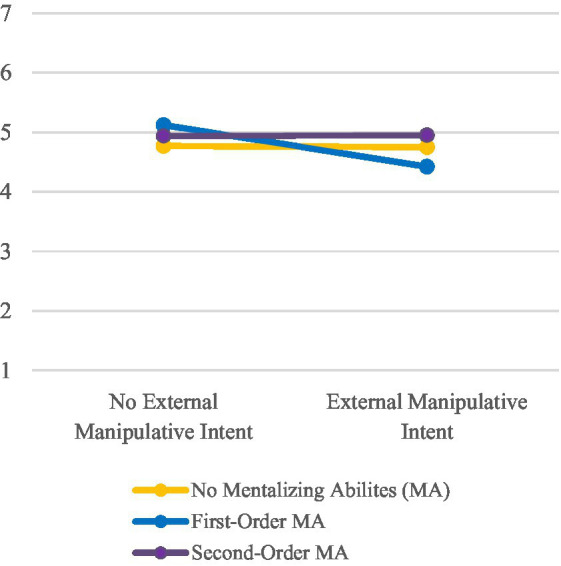
Mean values for participants’ attribution of trustworthiness to the robot.

Hypothesis 3 assumed an interaction effect between mentalizing abilities and external manipulative intent. Two significant interactions related to attributions of goodwill (*p* = 0.034, partial η^2^ = 0.03) and trustworthiness (*p* = 0.038, partial η^2^ = 0.03) were found during the calculated two-factor MANOVA (see Figures 5, 6). Attributions of goodwill and trustworthiness were slightly higher expressed in first-order than in no mentalizing conditions if the external manipulative intent was absent (inversely in conditions with external manipulative intent). In second-order mentalizing conditions, however, the mean values between conditions were almost similar. Thus, although the results slightly point in the direction of the hypothesis, the inconclusive interaction pattern forces us to dismiss Hypothesis 3.

### Hypothesis 4

Hypothesis 4 assumed a moderation effect of persuasion knowledge on the relationship between external manipulative intent and credibility, expected voucher fit, and purchase intention. Using the PROCESS v.4.1 macro (Hayes, 2017) we analyzed this relationship for each dependent variable. However, none of the analyzed moderation models showed a significant influence of people’s self-estimated persuasion knowledge on credibility or its related constructs competence (*p* = 0.469), goodwill (*p* = 0.343), trustworthiness (*p* = 0.296), expected voucher fit (*p* = 0.524), and buying attention (*p* = 0.349). Thus, like the previous investigation on the main effects, the anticipated moderation described in Hypothesis 4 receives no support from our experiment’s data (see Table 5 for an overview).

**Table 5:** Summary of results for the tests of hypotheses.

Hypotheses	Confirmed/Rejected
H1: Social Robots with theory of mind-abilities are rated more credible (indirectly measured [a] and explicitly measured [b]) than robots without ToM-abilities	Rejected (*p* > 0.05)
H2: External manipulative intent of an organization behind a robot leads to lower levels of attributed credibility for the robot (indirectly measured [a] and explicitly measured [b]) compared to a robot without external manipulative intent	Rejected (*p* > 0.05)
H3: Social robots with higher mentalizing abilities and external manipulative intent of a sponsoring company are rated least credible (indirectly measured [a] and explicitly measured [b])	Rejected (*p* > 0.05)
H4: Self-estimated persuasion knowledge moderates the external manipulative intent’s influence on people’s credibility ratings for the robot (indirectly measured [a] and explicitly measured [b])	Rejected (*p* > 0.05)

### Research questions

The first research question addressed the relationship between a robot’s alleged mentalizing abilities and users’ attributions of empathic understanding to the robot. An analysis of variance with mentalizing abilities as a single factor and emphatic understanding as dependent measure revealed significant differences between these three experimental conditions [*F* = (2,197) = 6.42, *p* = 0.002, η^2^ = 0.06; *M*_no mentalizing_ = 3.08, *M*_first-order mentalizing_ = 3.45, *M*_second-order mentalizing_ = 3.70]. Subsequent post-hoc tests revealed that especially second-order and no mentalizing conditions varied significantly, indicating that with a robot’s increasing mentalizing abilities participants associated higher empathic understanding with it. In addition, empathic understanding was (mediocrely) correlated with the study’s main dependent measures credibility competence (*r* = 0.41), goodwill (*r* = 0.62), trustworthiness (*r* = 0.48) as well as participant’s expected voucher fit (*r* = 0.25), and purchase intention (*r* = 0.26).

For the second research question, it was investigated whether the external manipulative intent caused any differences in participants’ sourceness perceptions related to the robot. We anticipated the robot to be regarded more as a medium than as the source of information if the voucher recommendation task was framed as being supported by a profit-oriented company. By using a Chi^2^-test to analyze the distribution of the given answers of the sentence-completion task, however, no significant differences could be found between conditions with or without external manipulative intent (*p* > 0.05, see Table 6 for an overview of the selected answers). Thus, no support for our anticipated difference in source perception was obtained.

**Table 6:** Distribution of given answers for the question who decided which voucher will be presented.

Question 3: “… made the decision which vouchers were presented to me.”		
	No ext. manipulative intent	Ext. manipulative intent
The robot	*n* = 85	*n* = 83
The university	*n* = 2	*n* = 3
The sponsor	*n* = 0	*n* = 2
Nobody	*n* = 2	*n* = 0

## Discussion

The study’s aim was to investigate how people’s evaluations of a robot with different mentalizing abilities change when confronted with a situation during which the robot is used for the interest of an organization with own financial interests. For this research aim, we designed a laboratory experiment during which we let 200 participants interact with a social humanoid robot as a recommender for experience vouchers. This interaction with the robot was either framed as the robot being used by a student research project or by a fictitious company recommending only its own experience vouchers *via* the robot.

In sum, we could not identify differences in credibility attributions toward the robot based on the hypothesized main effects of mentalizing abilities or external manipulative intent, neither regarding explicit measures nor indirect ones. Although the study successfully created the impression of an external manipulative intent, this setting did not cause differences in credibility ratings, expected product fit, participants’ purchase intention, or willingness to get help from the robot in a subsequent task. Similarly, the manipulation of the robot’s mentalizing abilities did not result in significant differences with respect to these measurements as well. Only a small interaction effect between mentalizing abilities and external manipulative intent was observed regarding the robot’s goodwill and trustworthiness. However, due to its inconclusive nature, this effect might be the product of chance and does not allow us to draw meaningful conclusions.

In contrast to the rejected main hypotheses, the first research question revealed a significant difference in attributed empathic understanding to the robot depending on the extent of its mentalizing abilities. However, no differences in terms of source perception induced by the external manipulative intent manipulation could be detected.

### Theoretical implications

As summarized before and contrary to previous studies (Sturgeon et al., 2019; Mou et al., 2020) no effects of a robot’s mentalizing abilities on credibility attributions were found. Despite a prior power analysis and more participants in single conditions compared to those previous experiments, we could not detect the assumed effects of credibility-supporting mentalizing abilities. However, it is important to consider that the manipulation of mentalizing abilities did cause differences in attributions of empathic understanding related to the robot, with higher abilities being related to higher attributions of empathic understanding. Most likely participants used the depiction of the robot’s mental processes as a reference for how much empathy they ascribe to it. Literature has pointed out that both these constructs are highly related, and their influence can be intertwined (Schurz et al., 2021), supporting the associative results found in this study. In addition, we observed moderate correlations between empathic understanding and explicit and indirect credibility attributions. On the one hand, these correlations are plausible as we assumed a robot’s ability to understand its users to affect credibility ratings, on the other hand, this partly contradicts the nonsignificant influence of mentalizing abilities on credibility we found as well.

A possible explanation for this result is, that the robot’s credibility was rather rated on interaction-based criteria (for example its choice of words or the fit of its reactions to participants’ answers) and not on the demonstration of its mentalizing abilities *via* text and picture stories prior to the interaction. It has been pointed out before that observing a robot’s actual behavior is of higher importance for evaluating it than secondary sources (Horstmann and Krämer, 2020). Hence, a live demonstration of the robot’s mentalizing abilities could assumably have had a greater effect on people’s credibility ratings and behavioral intentions. This might imply, that learning moderately important information about a robot before an interaction with it (especially if it’s for the first time), is a non-recommendable procedure to ensure that this information gets considered by users.

However, the studies by Sturgeon et al. (2019) and Mou et al. (2020) found an impact of mentalizing abilities on credibility using this procedure of prior demonstration *via* video. One reason for this difference to the current study could be the different types of interaction that followed the initial exposure to information about the robot. While the study conducted by Sturgeon et al. (2019) was an online experiment, that only included the manipulation videos and no interactive task, the study by Mou et al. (2020) additionally used a price estimation game as the main task where participants had to make several small price decisions to which the robot either agreed or disagreed. In contrast, our main task included a dialogue during which the robot asked the participants mostly open-ended questions (see Table 1). Thus, our interaction was less-repetitive and routinized, forcing participants to decide on their own what type of answer they give in terms of length, details, and complexity when the robot asked its questions (e.g., regarding the question “How athletic is your target person?” some participants simply answered by referring to the questions’ wording and said, “not athletic” or “very athletic,” while other participants explained in detail, what kind of sport their target person is doing currently in combination with details on frequency). Thus, this task was probably more attention-demanding task which could be a possible reason why the information learned beforehand did not become as relevant as during the study of Mou et al. (2020).

In addition, because this study used the Wizard-of-Oz technique, the robot’s answers to all questions were pre-scripted and thus identical and generally applicable to almost all participants’ answers. The robot always thanked the participants for each answer and continued with the next question, regardless of the answer’s length, detail, or complexity. Our intention was to keep the dialogue as similar as possible between all participants and still create realistic social interactions. However, participants most likely updated their mental model of the robot differently based on its reaction to their answers. If a participant delivered a complex answer and experienced the robot to understand it, this probably led to higher expectations of its abilities. On the other side, participants who gave the shortest possible answers only (e.g., “not athletic”) were less likely to form the same expectations of the robot’s abilities. For this reason, the study’s main task may have created too much unsystematic variance because it left too much room for different, unintentional experiences while using the robot. However, social interactions are far more complex than our scripted interaction. Therefore, the effect of prior information on a robot’s abilities might be even less effective in real-world scenarios, unless they are highly important for the interaction (such as safety information).

Regarding the manipulation of external manipulative intent, we did not detect participants to behave differently toward a social robot being associated with an either profit-or non-profit organization using it, even despite considering their general persuasion knowledge during our calculations. These results suggest again that participants blanked out background information when they engaged with the social robot and put a higher focus on the robot and the interaction itself than on the organization making use of it. As social robots are rarely seen in people’s everyday life (over 90% of participants did not interact with this robot before the study) this could have caused their attention to be mainly focused on the robot itself, despite each organization’s logo being continuously shown on the robot’s screen as a reminder of the manipulation. The higher focus on the robot would also help to explain why 84% of all participants considered the robot itself to have made the decision which vouchers were recommended (thus being seen as the source of information), independent from the manipulation.

Participants’ indifference in their reactions seems concerning regarding the influential potential of social robots as marketing tools. If people are equally likely to consider recommendations from a company or from non-profit organizations through the medium ‘robot’, this allows for manipulative advertising strategies. However, two counterarguments must be considered. First, participants did detect higher external manipulative intent when it was present in our study. Secondly, the salience of the organization was not as high as it would be during non-experimental human-robot interactions. The study took place in a neutral laboratory at a university and many of its students participated it (this argument receives support from the slightly higher mean values for the company’s manipulative intent during the pre-test compared to the laboratory study). People might pay more attention to the organization using the robot if the robot is used in the organization’s facilities (e.g., the store where the recommended products are sold) and the salience of the organization using the robot is therefore high.

In addition, no conclusive interaction effect was found, in line with our assumption that higher mentalizing abilities are becoming detrimental in situations with external manipulative intent. This is because, although there are small differences for example in trustworthiness between first-order mentalizing conditions with or without external manipulative intent, we cannot explain the non-appearance of this effect in no or second-order mentalizing conditions. Thus, the significant interaction effects regarding trustworthiness and goodwill do not allow to claim that either higher or lower mentalizing abilities are beneficial in situations with or without external manipulative intent. Especially due to the nonsignificant main effects, the low effect sizes, and small mean differences we conclude that these interaction effects are more likely the product of chance than signs of a meaningful interaction pattern.

### Limitations

One major limitation of our study is the fact that the robot’s comments on participants’ given answers during the voucher recommendation task were the same for each condition. Its sentences were created to be suited for every mentalizing condition, not given the participants any different information about the robot’s mental abilities than already shown before in the information texts and picture stories. However, this led to some minor problems during the conversation, e.g., the robot continuously referring to “the target person” even if participants mentioned their target person’s name or their relationship status. In such cases, the robot might have been not living up to the expectations of a robot that was shown to have a theory of mind.

Furthermore, the robot’s mentalizing abilities were only indirectly demonstrated by the information texts and picture stories. People had to guess the robot’s next behavior/thoughts in each picture story’s narrative based on the respective information text and could only continue the study when they made the right guess about what the robot is going to do/think (otherwise they had to answer each question again). Thus, our participants only indirectly learned about the robot’s abilities but did not see or experience the robot’s mentalizing behavior themselves. Additionally, the study took place in a university lab without a real company, real products to purchase and only limited consequences for participants to experience disadvantages from relying on the presented recommendations. Finally, the study’s convenience sample included majorly undergraduate female students which restricts us from drawing too general conclusions from our experiment.

### Conclusion

An experimental, laboratory study was conducted to investigate if social robots’ mentalizing abilities that have been shown to increase credibility attributions in past research can have opposite, detrimental effects on a robot if it is being used by an organization with manipulative intent. Contrary to our assumptions, statistical results suggest that mentalizing abilities only affected empathic understanding but neither explicit nor indirect credibility attributions to our humanoid robot. Additionally, despite a successful manipulation check, the external manipulative intent of an organization using the robot did not create any differences in participants’ evaluations or behavioral intentions related to the robot. Thus, in contrast to previous empirical findings, our research supports the assumption, that mentalizing abilities and credibility evaluations are not as closely related as expected. Furthermore, we did not detect a negative interaction effect when high mentalizing abilities and an external manipulative intent are present in combination. This lets us suggest that people rather consider primary information communicated by the robot than secondary meta-information about the organization behind it or the robot’s abilities.

## Data availability statement

The datasets (analyzed) for this study and its pre-study can be found in the OSF Repository (https://osf.io/pnmwd/).

## Ethics statement

The study, involving human participants, was reviewed and approved by the Ethics Committee of the Department of Computer Science and Applied Cognitive Science, Faculty of Engineering. Written informed consent to participate in this study was provided by each participant (and in one case the legal guardian). The individual(s) provided their written informed consent for the publication of any identifiable images or data presented in this article.

## Author contributions

MF conceptualized the experiment, collected the data, carried out the data analyses, and wrote the paper. NK supported the experiment by advising and revising during the whole process from conception to submission. All authors contributed to the article and approved the submitted version.

## Funding

The study only received internal financial support from the budget of the social psychology chair (University of Duisburg-Essen) where the experiment was conducted.

## Conflict of interest

The authors declare that the research was conducted in the absence of any commercial or financial relationships that could be construed as a potential conflict of interest.

## Publisher’s note

All claims expressed in this article are solely those of the authors and do not necessarily represent those of their affiliated organizations, or those of the publisher, the editors and the reviewers. Any product that may be evaluated in this article, or claim that may be made by its manufacturer, is not guaranteed or endorsed by the publisher.

## Footnotes

^1^ https://osf.io/pnmwd/
